# Prevalence of dental caries among a cohort of preschool children living in Gampaha district, Sri Lanka: A descriptive cross sectional study

**DOI:** 10.1186/1472-6831-12-49

**Published:** 2012-11-13

**Authors:** Priyantha J Perera, Nishadhi T Abeyweera, Meranthi P Fernando, Tania D Warnakulasuriya, Nayomi Ranathunga

**Affiliations:** 1 Faculty of Medicine, University of Kelaniya, Kelaniya, Sri Lanka; 2 North Colombo Teaching Hospital Ragama, Colombo, Sri Lanka; 3 Lady Ridgeway Hospital, Colombo, Sri Lanka

**Keywords:** Dental caries, Deft score, SIC index, Care index

## Abstract

**Background:**

Dental caries among young children are a global problem. Scant attention is paid towards primary teeth, leading to high prevalence of dental caries. There are only few studies done in Sri Lanka, addressing oral hygiene among preschool children. Scientific evidence is in need to persuade authorities to establish a programme promoting oral hygiene among preschool children.

**Methods:**

A descriptive cross sectional study was conducted in Ragama Medical officer of Health area. Consecutive children between 2 – 5 years of age, attending child welfare clinics were recruited for the study. Practices related to dental hygiene and socio-economic characteristics were obtained using an interviewer administered questionnaire. Mouth was examined for evidence of dental caries. Data collection and examination were done by two doctors who were trained for this purpose. The data were analysed using SSPS version 16.

**Results:**

Total of 410 children were included. None had a routine visits to a dentist. Practices related to tooth brushing were satisfactory. Prevalence of dental caries gradually increased with age to reach 68.8% by 5 years. Mean total decayed-extracted-filled (deft) score for the whole sample was 1.41 and Significant caries index (SIC) was 4.09. Decayed tooth were the main contributor for the deft score and Care index was only 1.55. Girls had a significantly higher prevalence of caries than boys.

**Conclusions:**

Dental care provided for Sri Lankan preschool children appears to be unsatisfactory as prevalence of dental caries among this cohort of preschool children was very high. There is an urgent need to improve dental care facilities for Sri Lankan preschool children.

## Background

Scant attention is generally paid towards protecting primary teeth, although they have many important functions. Apart from their role in feeding and phonation, they provide protection and space for permanent teeth [1]. Lack of care towards primary teeth, leads to poor oral hygiene and dental caries among preschool children. There are many well established public health programmes in Sri Lanka, but there is no such programme to promote oral health in preschool children.

Early childhood caries are a global public health problem. The American Academy of Paediatric Dentistry defines early childhood caries (ECC) as ‘the presence of caries in one or more primary teeth (cavitated or non-cavitated) in a child 71 months of age or younger’. ECC prevalence in England and USA is 6.8 - 12% and 11–53.1% respectively [2]. Highest prevalence of caries in maxillary anterior teeth is reported from Africa and South-East Asia [2]. In India, ECC prevalence among children between 8–48 months was reported as 44% [3].

Early stages of dental caries appear as white, chalky areas on the enamel close to the gum margin (enamel caries). As decaying process progresses to involve the dentine a visible cavity (dentinal caries) will develop. Once dental pulp is involved it becomes painful. Enamel caries are difficult to pick up, especially in uncooperative children. A mouth mirror and a good light source are needed to identify enamel caries. Mouth mirrors need sterilization in between patients and the main limiting factor in conducting dental research in a field setting is having adequate number of mouth mirrors. In contrast dentinal caries are easy to detect even without a mouth mirror.

Wrong feeding practices such as overnight feeding, adding sugar to formula milk and excessive consumption of sweets promote ECC [4,5]. In Sri Lanka a significant number of children are fed overnight, beyond the age of two years [6]. We often observe poor oral hygiene among preschool children, admitted to Teaching Hospital Ragama. Only few published reports are available on ECC among preschool children in Sri Lanka. Prevalence of ECC among 1–2 year olds was reported as 23% by one study [7], while the National Oral Health Survey 2002/03 reported a prevalence of 65% among 5 year olds [8]. Kumarihamy et al. (2011), reported an ECC prevalence of 32.19% in children aged 1–2 years [9].

This study was conducted with a view of describing the prevalence of dental caries among a cohort of Sri Lankan preschool children, living in Gampaha district, Sri Lanka.

## Methods

Sri Lanka is a low moderate income country, which has impressive health statistics. Maternal mortality rate (33.4 per 100,000) and infant mortality rate (10.1 per 1000 live births) in Sri Lanka, are among lowest in the region [10]. Ragama is situated in the Gampaha district, about 25 Km north of the capital, Colombo. In socio-economic standards, Gampaha is only second to Colombo district [11]. Ragama consists of a mixture of urban and suburban areas. The area belonging to Ragama Medical Officer of Health (MOH) extends over 25 Km^2^, and consists of a socio-economically mixed population. Child welfare clinics (CWC) are held twice a week within the Ragama MOH area. Immunization, nutritional assessment and growth monitoring are carried out at these clinics. According to Ragama MOH office, about 95% of children living in the area utilise facilities of these clinics.

A descriptive cross sectional study was carried out from August to October 2010, at CWCs held in Ragama MOH area. Reasons for selecting this area were the socioeconomically mixed population and easy accessibility. The study was based on guidelines from World Health Organization (WHO) on conducting dental research, with few modifications due to limitation in resources.

### Subject selection and training of investigators

From a preliminary survey, we found about 70% of preschool children admitted to Ragama Teaching Hospital had dental caries. A sample size of 323 was required to estimate a prevalence of 70% within 5% (prevalence of 70% and 95 confidence interval of 65%- 75%). According to the Ragama MOH office, in a month about 125 children attended CWCs. Children attending CWCs within a 3 month period were recruited for the study to ensure an adequate number in the sample.

Due to limited resources available at CWCs, only dentinal caries were studied. Data collection was by two pre-intern doctors who were trained to identify dental caries, by a consultant dental surgeon. Following the training, 35 children admitted to Ragama Teaching Hospital, but living outside the Ragama MOH area were examined by data collectors independently for dentinal caries. Kappa coefficient was one, which suggested very strong agreement between examiners. Restriction of the study to dentinal caries was the reason for strong agreement between examiners. Data collection process was supervised by the first author. Only children accompanied by the mother were recruited for the study. A child being included more than once, was prevented by questioning the mother about a previous examination. Informed written consent was obtained from the mother before recruitment. Children between 24 to 60 months of age, who attended clinics during the study period, were recruited on all-inclusive consecutive basis. None of the mothers refused to consent. Four hundred and forty one children were initially recruited, but examination could not be completed in 31 as the child was not cooperative.

### Data collection

An interviewer administered questionnaire asked about Socio-demographic characteristics, practices related to dental hygiene, history of dental diseases, dental fillings and visits to dentists. The questionnaire was validated and pretested on mothers admitted to Paediatrics units at Ragama hospital, but living outside the Ragama MOH area. Age of the child was defined as age in completed months.

Oral cavity was examined in the presence of the mother, under good light. A disposable wooden spatula was used whenever necessary. Mouth mirrors were not used because sterilization was not possible at CWCs and sufficient number of mirrors was not available for each child. This limited visualization, especially the upper molars but was adequate to detect or exclude dentinal caries. When food particles were present, mouth was rinsed for a better view. Both examiners were involved in examining each child to facilitate examination.

The deft score was calculated for each child by adding number of carious, extracted or filled teeth [12]. Significant Caries index (SIC) was calculated by adding the highest one third of deft scores and dividing it by one third of the total sample size [13]. The care index was deduced by, dividing the number of teeth with filling, by total number of teeth affected by caries and multiplying with 100. Only teeth with visible filling were considered under filled tooth.

### Ethical issues

A committee of experts from Sri Lanka College of Paediatricians reviewed the research proposal and Ethics Committee of Sri Lanka College of Paediatricians granted ethical approval for the study. Permission to conduct the study was obtained from the Medical Officer of Health, Ragama. Children with dental caries were referred to the dental clinic at Teaching Hospital, Ragama for treatment.

### Data analysis

The data were analysed using Statistical Package for Social Science (SPSS) version 16. Significance was set at, P < 0.05 (Significance level 95%). The prevalence of caries between different groups was compared with either ANOVA or Chi- square tests.

## Results

### Socio-demographic characteristics

Of the 410 children included in the study, 208 were males. Mean monthly family income of the study population was 22,580.49, Sri Lankan Rupees (One US dollar is equivalent to 130 Sri Lankan Rupees). Majority of mothers had an education between grades six to 11 (Sri Lankan students take the General Certificate of Education examination at grade 11, which is equivalent to GCSE in UK). Seventeen mothers (4.1%) had university education and one mother had not attended school.

### Practices related to oral hygiene

Majority of children (72.7%) had started tooth brushing before the age of one year and nearly all children (98.7%) had started brushing teeth by the age of two years. Three hundred and twenty nine children (80%) brushed teeth twice a day; 61(15%) once a day and the rest more than twice a day. Nearly all children (407) were using tooth paste. Of them 344 (84%) used a tooth paste containing fluoride and 34 (8%) used an Ayurvedic preparation. Nearly all children used a tooth brush. In 200 children (49%), parents brushed the child’s teeth, while 56 children (14%) brushed on their own. One hundred and fifty children (37%) brushed teeth under parent’s supervision.

To get treatment for dental diseases, 46 children (11%) had visited a dentist once, 13 (3%) had visited twice and 11 (3%) had visited more than twice. None had routine visit to a dentist.

### Prevalence and pattern of dental caries

Of the 410 children who were examined, 158 (38%) had at least one carious tooth at the time of examination or in the past. Prevalence of dental caries in relation to maternal education and family income is depicted in Table 1. Prevalence of dental caries had a statistically significant association with maternal education (p = 0.009), but the pattern was not of a steady decrease in caries with advanced maternal education. Children belonging to highest income group had the lowest prevalence of caries, but the middle income group had higher prevalence than lowest income group. The association between family income and prevalence of dental caries was not significant (p = 0.29).

**Table 1:** Prevalence of caries according to maternal education & family income

**Characteristics**	**Number of children (n = 410)**	**Number with caries**
**Mother’s education**		
None	1 (0.2%)	1 (100%)
Up to grade 5	5 (1.2%)	3 (60%)
Grade 6 to 11	247 (60.2%)	89 (36%)
Grade 12 to 14	140 (34.1%)	63 (45%)
University	17 (4.1%)	1 (5.8%)
**Income (Rs.)**		
<15000	111 (27.1%)	43 (38.7%)
15000-24999	148 (36.1%)	63 (42.6%)
> = 25000	151 (36.8%)	51 (33.8%)

Girls had a higher prevalence of caries (43.6%) than boys (33.7%), which was statistically significant (*χ*^2^ =4.239, p = 0.040). Prevalence of dental caries at 24 to 30 months was 8.9%. It increased to 68.8% by five years of age. Prevalence of dental caries according to the age of children is shown in Table 2.

**Table 2:** Prevalence of dental caries according to the age of children

Age*	**Number with caries**	**Prevalence**
24-29 months (n = 45)	04	08.9%
30-35 months (n = 80)	17	21.3%
36-41 months (n = 91)	27	29.7%
42-47 months (n = 63)	29	46.0%
48-53 months (n = 67)	37	55.2%
54-59 months (n = 64)	44	68.8%

Of the 158 children with dental caries, 125 (79.1%) had more than one tooth involved. Nineteen children (12%) had history of dental fillings according to the mother. Twelve children had a single tooth filled, six had two teeth filled and one child had three teeth filled. However, at the time of examination only five children and nine teeth had evidence of filling. The care index was 1.55.

Six children had tooth extractions due to dental caries. One had a single tooth extracted, while three had two teeth extracted and one each had six and seven teeth extracted respectively. Mean deft score for the total sample was 1.41, while mean deft scores for girls and boys were 1.72 and 1.12 respectively. The Sic index for total sample was 4.09, but the Sic index for children between 4 – 5 years of age was 5.84.

The deft score was calculated for each child. Majority of children had a score less than five, but there were children with scores of 10 and12 as well. Carious teeth made a major contribution towards the deft score. Distribution of the sample according to the deft score is shown in Table 3.

**Table 3:** Distribution of the sample according to deft scores

**Deft score**	**For girls**	**For boys**
0	114 (56.4)	138 (66.8%)
1	14 (6.9%)	19 (8.7%)
2	17 (8.4%)	14 (6.7%)
3	10 (5.0%)	11 (5.3%)
4	20 (9.9%)	9 (4.3%)
5	6 (3.0%)	5 (2.4%)
6	8 (4.0%)	5 (2.4%)
7	5 (2.5%)	2 (1.0%)
8	2 (1.0%)	2 (1.0%)
9	1 (0.5%)	0 (0.0%)
10	4 (2.0%)	2 (1.0%)
12	1 (0.5%)	1 (0.5%)

Maxillary incisors were the mostly affected by caries. Of the 581 caries observed, 271 (46.6%) involved them. High prevalence of caries was also noted in first molars of both jaws, with 186 (32.0%) caries present in these four teeth. Second molars in the lower jaw also had a high number of caries (72), but number of caries in second molars of the upper jaw was low (29). Other than the molars, teeth in lower jaw had only five carious teeth. Canines of the upper jaw had 17 carries, but the lower jaw had only two.

## Discussion

Although the primary dentition has many important functions, due to lack of care prevalence of dental caries is high among preschool children. According to American Academy of Dentistry, the first visit to a dentist should be at one year of age and all children should have at least two routine visits to a dentist each year. In this study population a large percentage of children had never been to a dentist. Few children have visited a dentist to get treatment for a dental problem. This confirms that preschool children in Sri Lanka do not get adequate dental care. This is in stark contrast to other impressive health indices of Sri Lanka. Results of this study emphasize the importance of strengthening community based dental care services for preschool children in Sri Lanka.

Prevalence of dental caries showed a gradual increase with age to reach 68.8%, by the age of five years. This is high compared to western countries like England (6.8 -12%) and USA (11–53.1%). According to Kumarihamy et al. (2011) prevalence of dental caries among Sri Lankan children between 1–2 years was 32.19% and the mean deft was 2.01. In our study we included only children between two to five years, as we were studying only dentinal caries. Inclusion of early stages of caries (enamel caries) by Kumarihamy et al., explains the higher deft score observed by them compared to us. Results of Sri Lanka National Oral Health Survey 2002/03, also revealed a high prevalence of caries (65%) at 5 year of age. Absence of a programme to promote oral hygiene among preschool children and wrong feeding practices may be contributing for the high prevalence of caries observed. Unfortunately no action is taken up to now, to promote oral hygiene among preschool children in Sri Lanka [8].

Although the SIC index for the total sample was 4.09, SIC index for children between 4 – 5 years was 5.84. WHO has a set a goal that globally the SIC index should be less than three, at 12 years of age, by year 2015 [12]. Although no target is set for preschool children, SIC index for Sri Lankan preschool children is nearly double the expected figure. When enamel caries are also included actual figures will be much higher than this.

In contrast to the findings of the United States National Health and Nutrition Examination Survey 1999–2004 [14], boys in this population had a lower prevalence of dental caries and a low mean deft score than girls. High individual deft scores were also recorded from girls than from boys. As there is no gender discrimination against female children in Sri Lanka, there must be other factors responsible for this gender difference in dental caries. Effect of gender on prevalence of dental caries is probably an important area for future research.

Not only primary prevention of caries was unsatisfactory, but the care for carious teeth was also unsatisfactory. Majority of the carious teeth were left unattended, allowing children subjected to pain, discomfort and premature loss of primary teeth. Very low care index (1.55) proves this fact. Our findings are compatible with findings of Kumarihamy et al., which revealed 95% of carious teeth were left unattended. Primary teeth are not generally extracted unless they cause significant discomfort and pain, as they keep space for the permanent teeth. In this study we found some children who had multiple tooth extractions, which is a terrifying and fearful experience for a child of this age.

Previous studies have shown children living in poverty have higher prevalence of caries (14), but a clear association between family income and dental caries was not observed in this study. Though the highest income group had lowest prevalence of caries, the middle income group had higher prevalence than the lower income group. The discrepancy may due to other confounding factors like feeding practices. Children of mothers with a low educational level had high prevalence caries and children of mothers with highest educational level had lowest prevalence. However, a reverse pattern was observed in the middle educational categories.

This study was conducted in a district with higher socio-economic standards compared to most of the districts in the country (11). Therefore, it is reasonable to postulate the situation in other parts of the country may be worse than this. There are effective programmes in Sri Lanka for promoting breast feeding, family planning, immunization, growth monitoring and school dental hygiene, but there is no programme to promote oral hygiene in preschool children. Even at child welfare clinics no attention is paid to oral hygiene. According to this study and the study by Kumarihamy et al., the process of dental decay commences at a very early age. Any educational or interventional programme aimed at promoting oral hygiene should be introduced around latter part of infancy.

Brushing teeth twice a day is an important mode of preventing dental caries. Effective brushing will remove dental plaques, which is the first step in dental decay. Using a tooth paste containing fluoride reduces decay by making enamel more resistant. In contrast to very high prevalence of dental caries in this population, tooth brushing was commenced at very young age and nearly all children were using a tooth paste containing fluoride. High prevalence of dental caries in the presence of good oral hygiene practices, points towards wrong feeding practices as the cause. A study conducted in the same area revealed a high prevalence of wrong feeding practices, like overnight feeding beyond two years of age and adding sugar to formula milk [6].

Pattern of dental decay described by this study is similar to findings of earlier studies [9]. Most commonly involved teeth were the maxillary incisors and these accounted for nearly half of the carious teeth. When children are fed at night, milk tends to pool between the upper lip and maxillary incisors. Carbohydrates in milk are fermented by bacteria leading to dental caries [15]. In contrast incisors of the lower jaw had a very low prevalence of caries, probably because tongue and lower lip cover them from pooled milk. In the lower jaw, molars in both quadrants had high prevalence of caries. In the upper jaw first molars had high prevalence of caries but second molars had relatively low prevalence, which may be due to late eruption. Canines were the least involved, probably because their shape providing some protection from caries.

### Limitations

The major limitation of this study was not using a dental mirror when examining teeth. Due to this only dentinal caries could be studied. The actual prevalence of dental caries will be higher than described by this study. The study setting was also not the ideal for this type of a study. Other limitation is, due to study population not representing the entire country finding of this study cannot be generalized as national figures.

## Conclusions

Prevalence of dental caries among preschool children in this population is very high. Majority of decayed tooth are left unattended, indicating poor dental care facilities for preschool children. Process of dental caries begins at very early age and progress steadily thereafter. An interventional program aimed at improving oral hygiene among preschool children is needed urgently. Although the prevalence of dental caries was high, practices related to tooth brushing were satisfactory, indicating wrong feeding practices as the possible cause.

## Competing interests

No funds were obtained to conduct this study and all authors declare that there are no competing interests.

## Authors’ contributions

PJP was involved in planning, supervising data collection, analysing data and preparing the manuscript. NTB was involved in planning and preparing the manuscript. MPF, NR and TDW were involved in planning, data collection, data analysing and helping in preparing the manuscript. All authors read and approved the final manuscript.

## Pre-publication history

The pre-publication history for this paper can be accessed here:

http://www.biomedcentral.com/1472-6831/12/49/prepub
